# Targeting Glycolysis May Bridge Innate and Adaptive Immune Regulation in Experimental Peri-Implantitis

**DOI:** 10.3390/ijms27177513

**Published:** 2026-08-22

**Authors:** Shudan Deng, Xingchen Liu, Feiyang Wu, Shoucheng Chen, Zhuofan Chen

**Affiliations:** 1 Hospital of Stomatology, Sun Yat-sen University, Guangzhou 510055, China; dengshd@mail2.sysu.edu.cn (S.D.); liuxch65@mail2.sysu.edu.cn (X.L.); wufy28@mail2.sysu.edu.cn (F.W.); 2 Guangdong Provincial Key Laboratory of Stomatology, Guangzhou 510055, China; 3 Guanghua School of Stomatology, Sun Yat-sen University, Guangzhou 510055, China; 4 Guangdong Research Center for Dental and Cranial Rehabilitation and Material Engineering, Guangzhou 510055, China

**Keywords:** peri-implantitis, 2-deoxy-D-glucose, immunomodulation, M1-associated markers, Th17-associated markers

## Abstract

Peri-implantitis is characterized by progressive tissue destruction accompanied by dysregulated innate and adaptive immune responses. This study evaluated whether systemic treatment with 2-deoxy-D-glucose (2-DG) could attenuate experimental peri-implantitis and alter inflammatory markers associated with macrophage- and T-cell-mediated responses in a rat model. Experimental peri-implantitis was induced in *Sprague–Dawley* rats by Porphyromonas gingivalis-inoculated silk ligation around the implant neck. After disease induction, animals received intraperitoneal injections of 2-DG or normal saline for six weeks while ligature-associated challenge was maintained. Peri-implant tissues were assessed by micro-computed tomography (micro-CT), hematoxylin and eosin staining, immunohistochemistry (IHC), and reverse transcription-quantitative PCR (RT-qPCR). 2-DG treatment significantly reduced peri-implant bone resorption and was associated with less pronounced bone-resorptive morphology. RT-qPCR showed lower expression of M1-associated genes (*CD86*, *iNOS*, *TNF-α*, *IL-6*, and *IL-18*) and the Th17-associated genes *IL-17* and *RORγT*, whereas *IL-21*, M2-associated genes, and the Treg-associated gene *FOXP3* were not significantly changed. IHC showed qualitative trends toward lower iNOS- and IL-17-positive signals. These findings provide proof-of-concept evidence that 2-DG treatment reduces peri-implant bone loss and is associated with attenuated inflammatory changes and lower expression of M1- and Th17-associated inflammatory markers in experimental peri-implantitis. This study supports further investigation of 2-DG as a potential immunometabolic adjunct for peri-implantitis management.

## 1. Introduction

Peri-implantitis is a major biological complication leading to late implant failure and has attracted increasing clinical attention. Epidemiological studies have reported a high prevalence of peri-implantitis, which may rise to 62.1% in implants with plaque accumulation [1]. With the continuous increase in implant therapy, the treatment, supportive maintenance, and defect reconstruction associated with peri-implantitis impose substantial costs and adversely affect patients’ perception of treatment outcomes and quality of life [2]. Studies have shown that the recurrence rate after treatment can reach up to 50% in some cohorts, and that baseline bone loss exceeding 50% may increase the risk of implant removal by 20-fold [3]. Despite continuous refinement of therapeutic approaches, clinical outcomes remain unsatisfactory, with limited bone regeneration and considerable implant loss [4,5]; the ITI Consensus Report notes that approximately one-quarter of affected implants may be lost within 5 years after therapy [4]. These unsatisfactory outcomes suggest that, beyond the persistence of microbial challenge, the host inflammatory microenvironment itself may act as a self-perpetuating driver of progressive tissue destruction and compromised regeneration.

Accumulating evidence indicates that the aggressive and refractory nature of peri-implantitis is associated with a complex inflammatory imbalance within the peri-implant microenvironment, involving both innate and adaptive immune responses [6,7]. Within the innate compartment, M1-associated macrophage programs promote pro-inflammatory mediators such as TNF-α, IL-1β, and IL-6, which can enhance osteoclastogenesis and osteoclast activity [8]. Within the adaptive compartment, Th17-associated responses can facilitate osteoclast differentiation through RANKL expression and IL-17-mediated induction of RANKL in local stromal and immune cells [9]. Crosstalk between macrophage- and T-cell-associated pathways may therefore sustain chronic inflammation, aggravate peri-implant soft- and hard-tissue destruction, and impair repair [7]. This coordinated inflammatory network may contribute to the progressive tissue destruction and treatment resistance observed in peri-implantitis.

However, simultaneously modulating innate- and adaptive-immune-associated inflammatory pathways remains challenging. Conventional immunomodulatory approaches commonly focus on a single cell population or signaling axis. For example, miRNA-releasing implant coatings have been designed to reprogram macrophage mitochondrial metabolism and polarization [10], whereas CCR2-targeted strategies primarily restrict inflammatory monocyte/macrophage recruitment [11]. Although such approaches can be effective within their intended axis, they may not fully address the extensive crosstalk between macrophage- and T-cell-mediated responses. A strategy that acts on a metabolic requirement shared by multiple pro-inflammatory programs could therefore provide a complementary approach.

Immunometabolic studies suggest that activated M1 macrophages and Th17 cells often increase glucose uptake and glycolytic flux through pathways involving mTOR- and HIF-1α-associated metabolic reprogramming [12,13,14,15,16,17,18]. Increased glycolysis supplies rapid ATP and biosynthetic intermediates and supports transcriptional and cytokine programs associated with inflammatory effector functions. By contrast, M2 macrophages and Treg cells generally rely more strongly on mitochondrial oxidative metabolism and may be less sensitive to partial glycolytic restriction [12,13,14,15,16,17,18]. 2-DG is a glucose analogue that competes with glucose and accumulates as a poorly metabolizable phosphorylated intermediate, thereby limiting glycolytic flux. Previous studies have reported that 2-DG can reduce M1- and Th17-associated responses under selected experimental conditions while having more limited effects on M2- and Treg-associated programs [13,14,15,16,17,18]. These observations identify glycolysis as a plausible, but not yet proven in vivo, convergent regulatory mechanism in peri-implantitis.

Accordingly, this study used a rat model of experimental peri-implantitis to evaluate whether systemic 2-DG treatment could attenuate peri-implant bone loss and modulate inflammatory markers related to M1/M2 macrophage polarization and Th17/Treg responses in peri-implant tissues. We further explored the potential of targeting glycolysis with 2-DG as an immunometabolic strategy for modulating innate- and adaptive-immunity-associated inflammatory responses in peri-implantitis.

## 2. Results

### 2.1. Histological Overview of Peri-Implant Soft and Hard Tissue Differences

**Figure 1:**
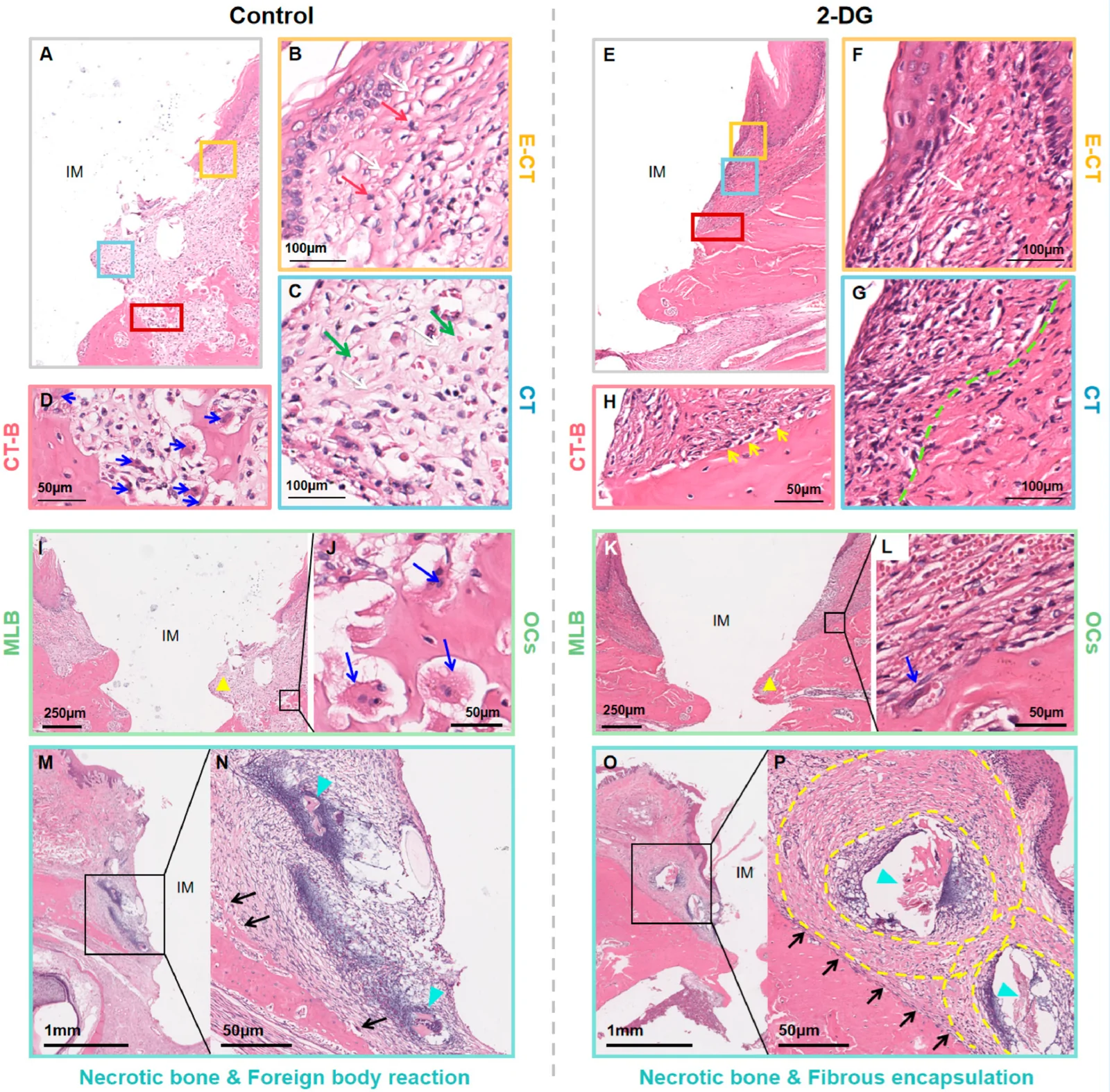
Histological evaluation of peri-implant soft tissue and marginal bone morphology. (**A**–**H**) Regional evaluation from the coronal to apical direction. Yellow, blue, and red boxes in (**A**,**E**) correspond to the enlarged subepithelial (**B**,**F**), middle connective-tissue (**C**,**G**), and soft tissue-bone interface (**D**,**H**) regions, respectively. Red arrows indicate inflammatory-cell clusters; white arrow in (**B**,**C**) indicates wider interfibrillar spaces, while the white arrow in (**F**) indicates more densely packed collagen bundles; green arrows indicate erythrocytes; the green dashed line marks the approximate boundary between localized altered tissue and more compact collagen. (**D**) Blue arrows indicate osteoclast-like multinucleated cells or resorption lacunae. (**H**) Yellow arrows indicate cells aligned along the bone surface. (**I**–**L**) Yellow triangles mark the marginal bone level adjacent to the first implant thread. Blue arrows indicate osteoclast-like multinucleated cells or resorption lacunae. (**M**–**P**) Cyan arrowheads indicate necrotic bone-like material; black arrows indicate adjacent inflammatory/foreign-body-associated cellular reaction; yellow dashed lines outline a fibrous boundary. IM, implant space; E-CT, epithelial-connective tissue region; CT, connective tissue; CT-B, connective tissue-bone interface; MLB, marginal bone level; OCs, osteoclast-like cells. Scale bars are shown in each panel. Representative morphology is descriptive; no inferential statistical test was applied.

Decalcified paraffin sections from the control group and the 2-DG group were stained with hematoxylin and eosin (H&E) and examined from the coronal to apical direction. Representative sections showed inflammatory alterations in peri-implant soft and hard tissues in both groups (Figure 1A–H). At the bone interface, irregular bone surfaces, resorption lacunae, and osteoclast-like multinucleated cells were more conspicuous in representative control sections than in representative 2-DG sections (Figure 1D,H).

For regional evaluation, the peri-implant tissues were divided into three representative areas: the subepithelial region, the middle connective-tissue region, and the soft tissue-bone interface (Figure 1A–H). The color-matched boxes in the overview images indicate the regions enlarged in the corresponding panels. In the subepithelial region, representative control sections showed wider interfibrillar spaces and more conspicuous inflammatory-cell clusters (white and red arrows, respectively; Figure 1B), whereas the corresponding 2-DG region showed relatively more compact collagen bundles (white arrows; Figure 1F) outside areas of residual cellular infiltration.

In the middle connective-tissue region, the representative control section showed loosely arranged collagen, accompanied by diffuse erythrocyte extravasation (white and green arrows respectively; Figure 1C). In the 2-DG section, tissue alteration and cellular infiltration were more localized near the implant-facing region, while collagen bundles beyond the green dashed boundary appeared relatively compact (Figure 1G). Because these features were assessed qualitatively, the images should not be interpreted as demonstrating complete normalization of soft tissue.

At the soft tissue-bone interface, the control section showed an uneven bone surface with resorption lacunae and osteoclast-like multinucleated cells (blue arrows; Figure 1D). The 2-DG section showed a comparatively smoother interface, fewer conspicuous lacunae, cells aligned along the bone surface (yellow arrows), and a partially continuous periosteum-like boundary (Figure 1H). These annotations identify the morphological features described in the text but do not constitute quantitative cell counts.

Representative low- and high-magnification images further suggested differences in marginal bone morphology (Figure 1I–L). Yellow triangles mark the marginal bone level adjacent to the first implant thread. The control section showed a lower marginal bone level and less bone contact in the corresponding region (Figure 1I,J), whereas the 2-DG section showed comparatively better preservation of the bone contour (Figure 1K,L). Blue arrows indicate osteoclast-like cells or resorption lacunae. Hard-tissue sections from the 2-DG group are additionally shown in Figure S1.

Necrotic bone-like areas were observed in representative sections from both groups (Figure 1M–P). Cyan arrowheads indicate necrotic bone-like material. In the control section, black arrows indicate a dense cellular reaction extending into adjacent connective tissue without a clearly demarcated capsule (Figure 1M,N). In the 2-DG section, the necrotic bone-like area was surrounded by a more clearly defined fibrous boundary (yellow dashed outlines), with adjacent cellular reaction indicated by black arrows (Figure 1O,P). Collectively, these qualitative observations were consistent with less extensive inflammatory and bone-resorptive morphology in the 2-DG group, but they do not demonstrate restoration to healthy peri-implant tissue.

### 2.2. 2-DG Reduces Peri-Implant Bone Resorption

**Figure 2:**
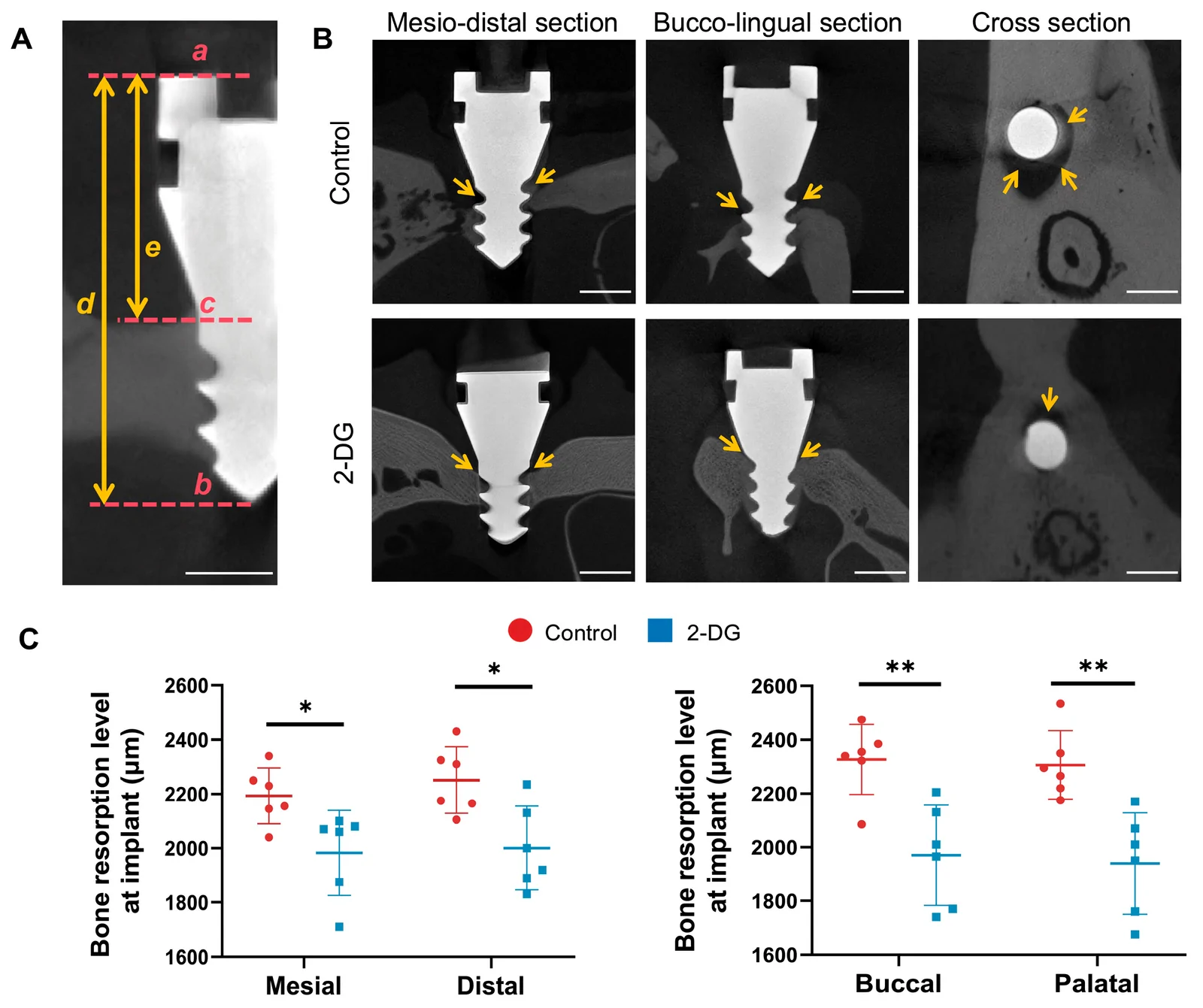
Micro-CT assessment of peri-implant bone resorption. (**A**) Geometric correction method: exposed implant height (mm) = 3.8 × (e/d), where e is the measured exposed length in the section and d is the implant length captured in the same plane; scale bars, 1 mm. (**B**) Representative mesiodistal, buccolingual, and cross-sectional reconstructions. Yellow arrows indicate the most coronal bone crest/bone-to-implant contact; scale bars, 1 mm. (**C**) Bone-resorption measurements at the mesial, distal, buccal, and palatal aspects. Data are presented as mean ± SD and were compared using an unpaired two-tailed Student’s *t*-test. * *p* < 0.05; ** *p* < 0.01.

Micro-CT was performed on implants using standardized mesiodistal and buccolingual sections. Peri-implant bone resorption was quantified by measuring the exposed implant length above the most coronal bone-to-implant contact at the mesial, distal, buccal, and palatal aspects. Because the enlarged implant neck served as a standardized insertion stop, greater exposed implant length indicated greater bone loss (Figure 2A). Representative reconstructions showed less bone support around control implants than around 2-DG-treated implants; yellow arrows identify the most coronal bone crest/bone-to-implant contact used for interpretation (Figure 2B). Quantitative analysis showed significantly lower bone-resorption measurements in the 2-DG group at all four aspects (Figure 2C).

### 2.3. Histomorphometric Analysis of Peri-Implant Soft Tissue

**Figure 3:**
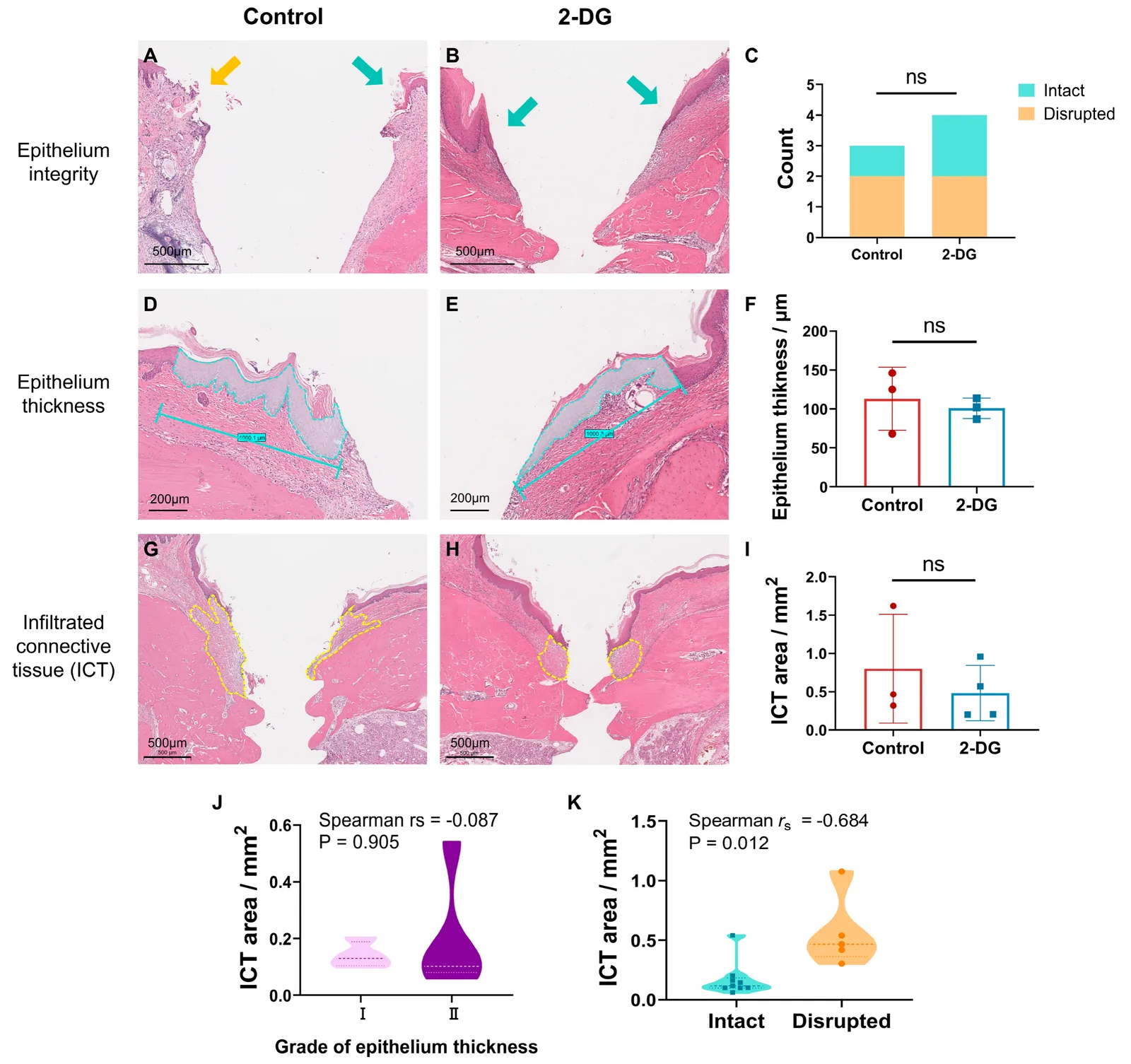
Quantitative histological analysis of peri-implant soft tissue. (**A**–**C**) Representative images and comparison of epithelial integrity. Green arrows, intact epithelium; orange arrow, disrupted epithelium. (**D**–**F**) Epithelial-thickness measurement; cyan outlines indicate the measured epithelial area and reference length. (**G**–**I**) ICT measurement; yellow dashed lines outline the ICT region. (**J**,**K**) Spearman correlation analyses. Epithelial integrity was compared using the Mann–Whitney U test, epithelial thickness and ICT area using unpaired two-tailed Student’s *t*-tests, and associations using Spearman correlation. Data in (**F**,**I**) are mean ± SD; ns, not significant.

H&E-stained sections were analyzed for epithelial integrity, epithelial thickness, and infiltrated connective-tissue (ICT) area using the criteria described in Section 4.7. Green arrows identify intact epithelium, the orange arrow identifies disrupted epithelium, cyan outlines indicate the epithelial area used to calculate average thickness, and yellow dashed lines outline the ICT region (Figure 3A–H). The distribution of intact versus disrupted epithelium did not differ significantly between groups (Figure 3C), and epithelial thickness was also comparable (Figure 3D–F).

The ICT area tended to be smaller in the 2-DG group, but the difference was not statistically significant (Figure 3G–I). Epithelial thickness was not significantly associated with ICT area (Spearman r_s = −0.087, *p* = 0.905; Figure 3J), whereas disrupted epithelial integrity was associated with a larger ICT area (Spearman r_s = −0.684, *p* = 0.012; Figure 3K). Thus, several quantitative soft-tissue parameters remained similar between the two inflamed groups, and the present data do not establish restoration toward a healthy peri-implant reference state.

### 2.4. Molecular and Immunohistochemical Assessment of Immune-Associated Markers in Peri-Implant Soft Tissue

**Figure 4:**
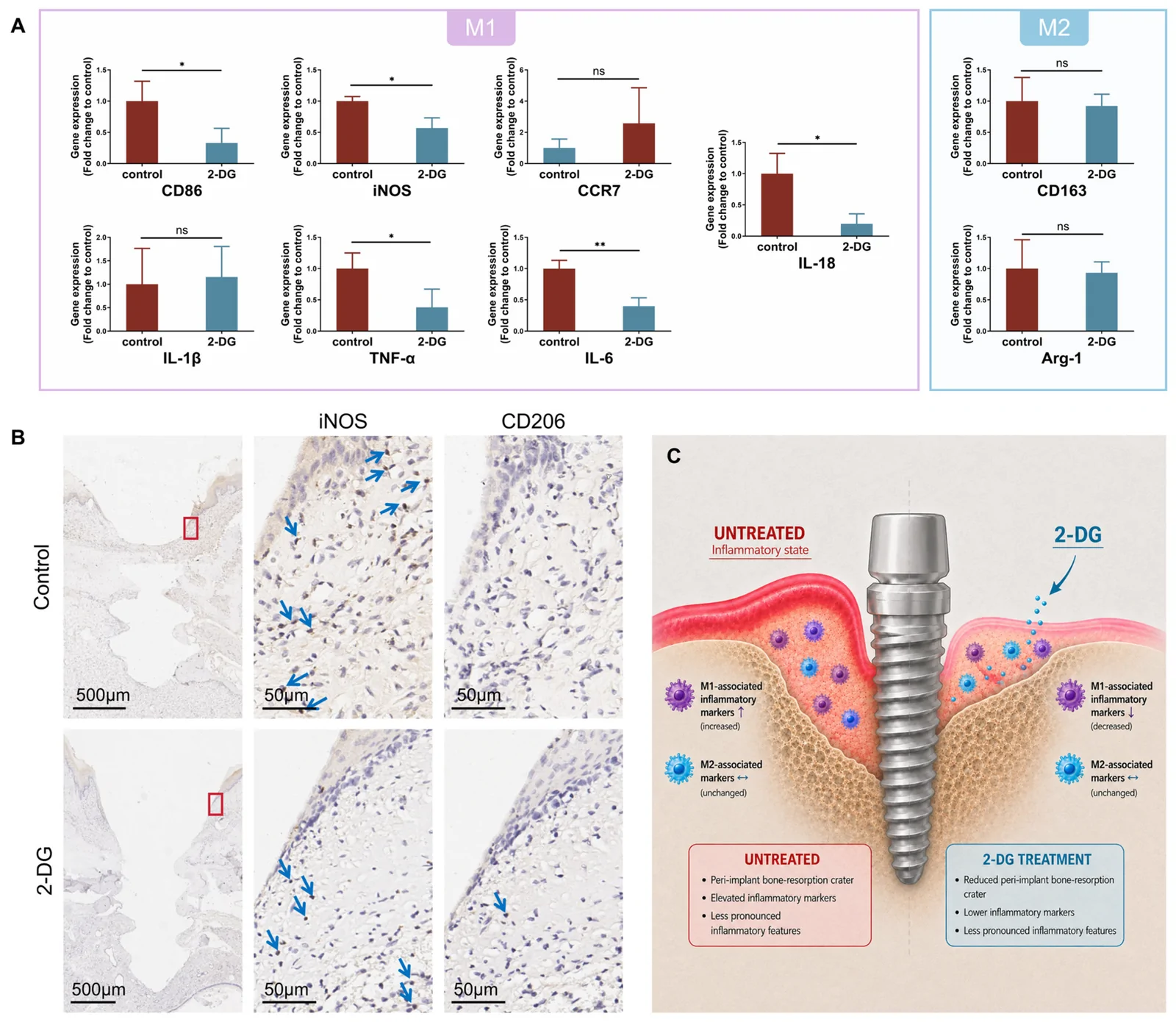
2-DG treatment is associated with reduced M1-related inflammatory markers in peri-implant soft tissue. (**A**) Whole-tissue RT-qPCR analysis. *CD86*, *iNOS*, *TNF-α*, *IL-6*, and *IL-18* were significantly lower in the 2-DG group; *CCR7*, *IL-1β*, *CD163*, and *Arg-1* were not significantly different and are labeled ns. Data are mean ± SD and were compared using unpaired two-tailed Student’s *t*-tests. * *p* < 0.05; ** *p* < 0.01; ns, not significant. (**B**) Representative IHC staining for iNOS and CD206. Blue arrows indicate representative positive staining; red boxes identify the enlarged regions. IHC was interpreted qualitatively and was not used to quantify macrophage abundance. (**C**) Schematic summary of marker-expression trends; the icons do not represent measured immune-cell counts.

**Figure 5:**
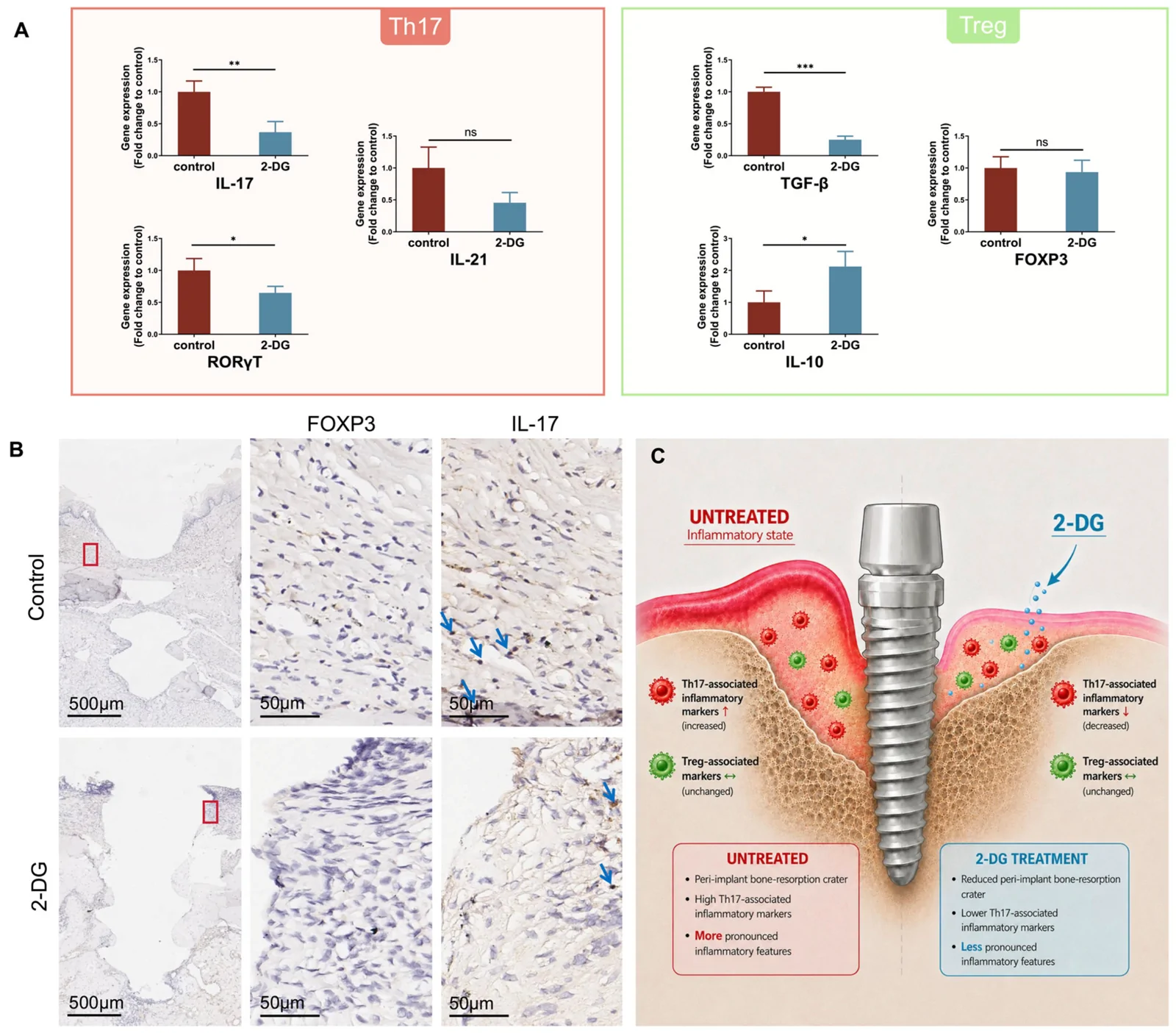
2-DG treatment is associated with reduced Th17-related inflammatory markers in peri-implant soft tissue. (**A**) Whole-tissue RT-qPCR analysis of Th17- and Treg-associated genes. *IL-17*, *RORγT*, and *TGF-β* were lower; *IL-10* was higher; *FOXP3* and *IL-21* were unchanged. Data are mean ± SD and were compared using unpaired two-tailed Student’s *t*-tests. * *p* < 0.05; ** *p* < 0.01; *** *p* < 0.001; ns, not significant. (**B**) Representative IHC staining for FOXP3 and IL-17. Blue arrows indicate representative positive staining; red boxes identify enlarged regions. IHC was interpreted qualitatively, and FOXP3-positive cells were scarce in both groups. (**C**) Schematic summary of marker-expression trends; the icons do not represent measured immune-cell counts.

Peri-implant soft tissues were analyzed by RT-qPCR for macrophage- and T-cell-associated genes, with expression normalized to GAPDH. Consecutive paraffin sections from corresponding regions were stained by IHC for iNOS/CD206 and IL-17/FOXP3 (Figure 4 and Figure 5).

Whole-tissue RT-qPCR showed significantly lower expression of the M1-associated genes *CD86*, *iNOS*, *TNF-α*, *IL-6*, and *IL-18* in the 2-DG group (Figure 4A). *CCR7* and *IL-1β* did not differ significantly. The M2-associated genes *CD163* and *Arg-1* also showed no significant between-group differences. Representative IHC images showed a qualitative trend toward lower iNOS-positive staining in the 2-DG group, whereas CD206 staining showed no obvious visual difference (Figure 4B). Together, these findings indicate that 2-DG treatment was associated with a reduced M1-associated inflammatory signature in peri-implant soft tissues. Figure 4C schematically summarizes the observed marker-expression trends.

For T-cell-associated markers, whole-tissue RT-qPCR showed significantly lower expression of *IL-17* and *RORγT* in the 2-DG group, whereas *IL-21* showed no significant between-group difference (Figure 5A). Among Treg-associated readouts, *TGF-β* was lower, *IL-10* was higher, and *FOXP3* showed no significant difference. Because TGF-β and IL-10 can be produced by multiple cell types, their divergent changes cannot be interpreted as direct evidence of altered Treg abundance or function. Representative IHC images showed a qualitative trend toward lower IL-17-positive staining in the 2-DG group, whereas FOXP3-positive cells were scarce in both groups (Figure 5B). Figure 5C summarizes these marker-expression trends schematically.

Taken together, the molecular data indicate that 2-DG treatment was associated with lower M1- and Th17-related inflammatory signatures in peri-implant soft tissue, while M2-associated genes and FOXP3 were not significantly altered. These findings should not be interpreted as direct evidence that specific immune-cell populations were selectively depleted or functionally reprogrammed.

## 3. Discussion

### 3.1. 2-DG Is Associated with Attenuated Inflammatory and Bone-Resorptive Outcomes

Healthy peri-implant connective tissue is generally characterized by dense collagen organization, limited vascularity, and minimal inflammatory-cell infiltration [19,20]. In the present study, both the control and 2-DG groups retained inflammatory histological features, confirming that 2-DG did not eliminate the induced disease state. Representative sections suggested less extensive tissue loosening and bone-resorptive morphology after 2-DG treatment; however, epithelial integrity, epithelial thickness, and ICT area did not differ significantly between groups. These results indicate a relative treatment effect between two inflamed conditions rather than demonstrated restoration to physiological peri-implant tissue.

Human peri-implantitis lesions contain heterogeneous leukocyte populations, including neutrophils, plasma cells, T cells, B cells, and macrophages [20,21,22,23,24,25,26,27]. Although cell morphology in H&E sections may suggest the presence of particular leukocyte types, H&E staining alone cannot reliably assign immune-cell lineage or functional phenotype. Accordingly, the present histological observations were interpreted at the level of tissue morphology and inflammatory cellularity rather than as quantitative evidence for changes in specific leukocyte populations.

Micro-CT provided the strongest quantitative evidence of treatment-associated benefit: bone-resorption measurements were significantly lower on the mesial, distal, buccal, and palatal aspects in the 2-DG group. Osteoclast-like cells and resorption lacunae appeared more conspicuous in representative control sections, but osteoclast number or activity was not quantified by TRAP staining, histomorphometry, or functional assays. Therefore, the data support reduced bone resorption but do not directly demonstrate reduced osteoclast formation. Lower expression of inflammatory mediators such as TNF-α, IL-6, IL-17, and IL-18 provides a plausible route through which the local environment could influence RANKL-dependent osteoclastogenesis [28,29,30,31,32], but this relationship remains inferential.

### 3.2. Interpretation of Immune-Associated Marker Changes and Comparison with Previous Studies

CD86 and iNOS are commonly used as M1-associated markers, whereas CD163 and Arg-1 are commonly used as M2-associated markers [33,34]. In the present whole-tissue RT-qPCR analysis, CD86, iNOS, TNF-α, IL-6, and IL-18 were lower after 2-DG treatment, while CCR7 and IL-1β were unchanged. CD163 and Arg-1 were also unchanged. These patterns are consistent with attenuation of selected M1-associated inflammatory programs, but whole-tissue RNA cannot determine whether the changes resulted from altered macrophage abundance, polarization, transcription per cell, or contributions from other cell types. The qualitative IHC trend for iNOS was concordant with the transcript data but did not provide quantitative confirmation.

Similarly, the lower expression of IL-17 and RORγT, together with unchanged IL-21, is consistent with attenuation of a Th17-associated inflammatory program. FOXP3 was unchanged, and FOXP3-positive cells were rarely detected by IHC. The opposite changes in TGF-β and IL-10 should be interpreted cautiously because both cytokines can be produced by multiple immune and stromal cell populations and therefore do not specifically report Treg abundance or function. Cell-resolved methods, such as flow cytometry, multiplex immunofluorescence, or single-cell transcriptomics, will be required to establish whether 2-DG changes the number, phenotype, or activity of macrophage and T-cell subsets in peri-implantitis.

The present findings are broadly consistent with prior oral immunometabolic studies but differ in experimental resolution and delivery route. An earlier in vitro study on debrided titanium surfaces reported that 2-DG altered macrophage- and T-cell-associated immune readouts [18]. More recently, Zhao et al. used a thermosensitive PLGA-PEG-PLGA hydrogel to deliver 2-DG locally in murine periodontitis and, using cell-resolved assays, reported suppression of CD4+ T-cell expansion and Th17 polarization together with reduced periodontal inflammation and bone loss [35]. The current study extends the investigation to systemic 2-DG treatment in a rat peri-implantitis model and evaluates both M1- and Th17-associated tissue signatures. Conversely, it lacks the direct metabolic and cell-specific measurements available in the periodontitis study, so mechanistic conclusions should remain more limited.

Activated M1 macrophages and Th17 cells often rely on increased glycolytic flux, providing a biologically plausible explanation for the parallel reduction in selected M1- and Th17-associated markers [12,13,14,15,16,17,18]. Nevertheless, glycolytic activity was not measured in the present tissues, and 2-DG can affect multiple cell types and pathways beyond a single immune-cell subset. Glycolysis should therefore be regarded here as a working hypothesis for a convergent regulatory mechanism rather than a demonstrated causal bridge between innate and adaptive immunity.

### 3.3. Limitations and Translational Considerations

Several limitations should be considered. First, no healthy implant/non-peri-implantitis reference group was included. The present experiment therefore provides only a two-arm comparison between 2-DG and normal-saline vehicle after disease establishment. Consequently, the study cannot determine whether 2-DG restored inflammatory markers toward physiological values, and the nonsignificant differences in several soft-tissue parameters emphasize that both groups remained inflamed. Second, RT-qPCR was performed on whole peri-implant soft tissue, and IHC was qualitative, so cell number, polarization state, and functional activity were not directly established. Third, glycolytic flux and related metabolic intermediates were not measured. Fourth, osteoclast formation was not quantitatively assessed. Finally, the small exploratory sample, systemic treatment route, and absence of dedicated systemic safety measurements limit immediate translational interpretation.

Future studies should include healthy/non-ligated reference implants, cell-resolved immune profiling, direct measurements of glycolytic activity, quantitative osteoclast analyses, and systemic safety monitoring. Local sustained delivery through hydrogels or implant coatings may reduce systemic exposure while increasing peri-implant drug availability, as suggested by recent local-delivery work in periodontitis [35]. Dose, release kinetics, route, and potential off-target effects will require systematic optimization before 2-DG can be considered as a clinical adjunct.

## 4. Materials and Methods

### 4.1. Animals

Eight male Sprague–Dawley rats (450–500 g, 12–13 weeks old) were purchased from Guangdong Medical Laboratory Animal Center (Guangzhou, China). The animals were housed in a specific pathogen-free barrier facility. All experimental procedures were approved by the Institutional Animal Care and Use Committee of Sun Yat-sen University (approval No. SYSU-IACUC-2023-000210) and conducted in accordance with established animal use protocols.

### 4.2. Implant Design

**Figure 6:**
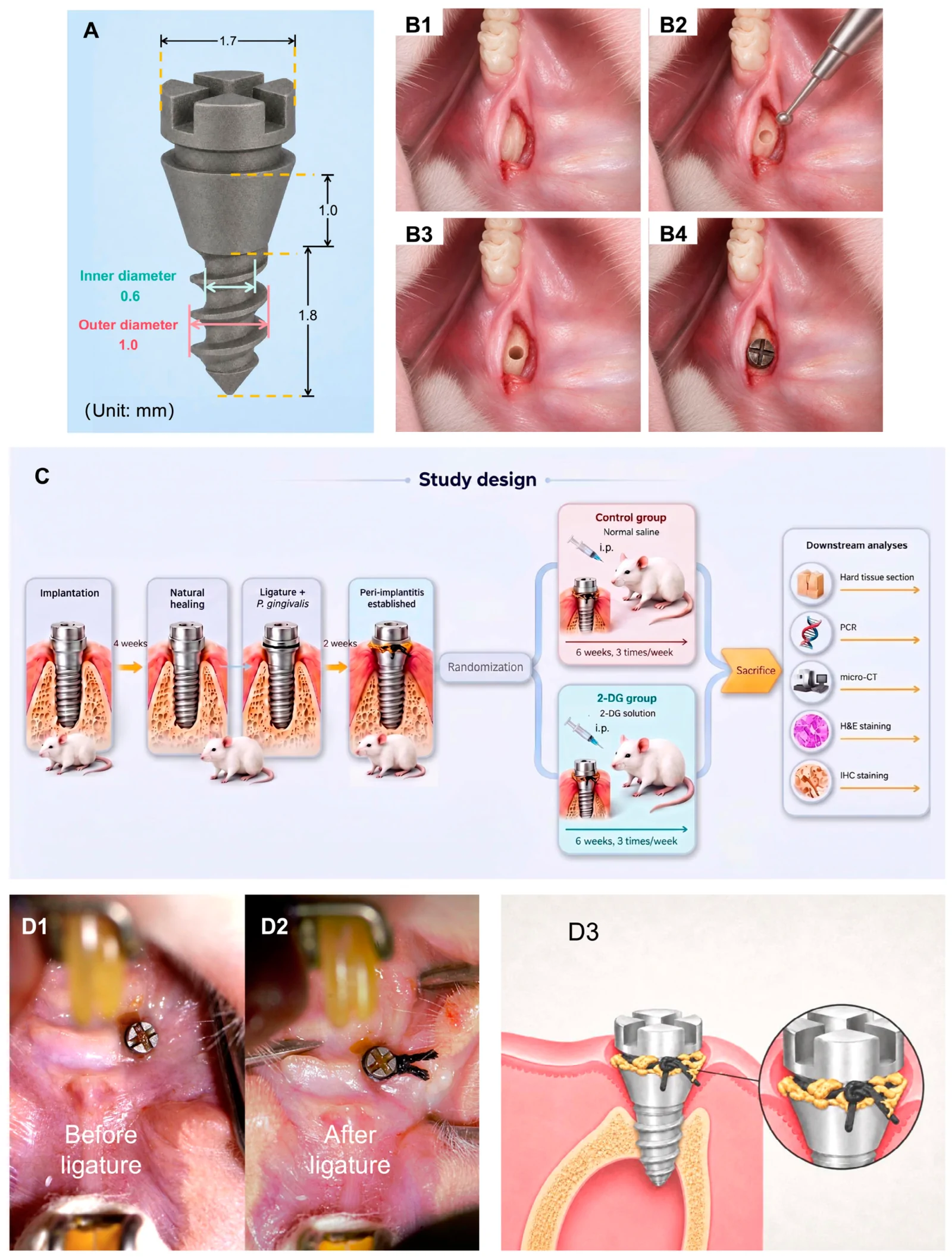
Implant design and establishment of the experimental peri-implantitis model. (**A**) Dimensions of the customized implant (unit: mm). (**B1**–**B4**) Surgical sequence: incision and flap elevation, round-bur positioning, sequential osteotomy, and implant placement. (**C**) Experimental timeline. After randomization, the control group received normal saline intraperitoneally and the 2-DG group received 2-DG (500 mg/kg) intraperitoneally three times weekly for six weeks; no local peri-implant injection was performed. (**D1**,**D2**) Clinical photographs before and after silk ligation. (**D3**) Schematic of silk ligation and plaque accumulation around the implant neck.

The implant geometry was customized to the rat maxilla and followed the previously standardized transmucosal design [36]. The implants (Shanghai Lingjian Precision Machinery Co., Ltd., Shanghai, China) were fabricated from Ti-6Al-4V. The overall physical length used for calibration was 3.8 mm; the coronal head width was 1.7 mm, the transmucosal collar height was 1.0 mm, the intraosseous threaded segment was 1.8 mm, the outer threaded diameter was 1.0 mm, and the inner/cervical diameter was 0.6 mm (Figure 6A). The surface was sandblasted with Al_2_O_3_ particles (110 µm, 280 kPa, 10 s) and acid-etched with HCl (pH 3, 80 °C, 20 min) to generate a rough surface.

### 4.3. Surgery

Before surgery, rats received subcutaneous atropine (Tianjin Jinyao Pharmaceutical Co., Ltd., Tianjin, China) to reduce salivary secretion. General anesthesia was induced by intraperitoneal Zoletil (Virbac S.A., Carros, France), consisting of tiletamine hydrochloride and zolazepam hydrochloride at a 1:1 mass ratio, and animals were positioned supine. The No. 15 surgical blade (Swann-Morton Ltd., Sheffield, UK) was used to make an incision parallel to the dental arch, approximately 2 mm mesial to the transgingival point of the maxillary first molar and slightly toward the palatal side to avoid major buccal vessels. A limited mucoperiosteal flap was elevated to expose the maxillary surface. The implant site was positioned 4–5 mm mesial to the transgingival point (Figure 6(B1)). A 0.5-millimeter round bur (Komet, Lemgo, Germany; 005) created the initial concavity (Figure 6(B2)), followed by sequential osteotomy under continuous 4 °C saline irrigation using 0.5-, 0.7-, and 0.85-millimeter twist drills (HROCHER TOOL, Shanghai, China) (Figure 6(B3)). Implants were manually inserted until the lower edge of the enlarged cervical stop was level with the bone surface (Figure 6(B4)). Implants were placed bilaterally, with one implant in the left and one in the right anterior maxilla of each surviving rat.

Eight rats underwent surgery; one animal died intraoperatively, and 14 implants were successfully placed in the seven surviving animals. Postoperative medication was administered once daily for three consecutive days: penicillin (North China Pharmaceutical Co., Ltd., Shijiazhuang, China) for infection prophylaxis and Antondine (Kunming Pharmaceutical Group Co., Ltd., Kunming, China) for analgesia. Animals received a soft diet for four weeks to permit osseointegration.

### 4.4. Bacterial Culture

Porphyromonas gingivalis ATCC 33277 was cultured on modified Brain Heart Infusion agar (Sigma-Aldrich, St. Louis, MO, USA) supplemented with 5–10% defibrinated sheep blood (Solarbio, Beijing, China; Cat. No. TX0030). Cultures were maintained in an anaerobic chamber (Don Whitley Scientific Ltd., Bingley, West Yorkshire, UK) at 37 °C and pH 7.2–7.4 for 48–72 h. Bacteria were harvested during logarithmic growth and standardized to 1 × 10^10^ CFU/mL.

### 4.5. Induction of Peri-Implantitis and 2-DG Intervention

The experimental timeline is shown in Figure 6C. Four weeks after implantation, implants with satisfactory soft-tissue healing and no redness, bleeding, or clinical mobility were eligible for disease induction (Figure 6(D1)). Among the seven surviving rats, 13 implants were randomly allocated to the control group (6 implants in 3 rats) or the 2-DG group (7 implants in 4 rats). Sterile 4-0 silk sutures (Ethicon, Somerville, NJ, USA; Cat. No. D9747) were immersed in the P. gingivalis suspension and tied around the implant neck to promote plaque accumulation (Figure 6(D2,D3)). A separate healthy/non-ligated sham group was not included. This design limitation is addressed in Section 3.3.

A 125 mg/mL 2-DG stock solution (MedChemExpress, Shanghai, China) was prepared and stored at −20 °C. Two weeks after ligation, the 2-DG group received intraperitoneal 2-DG at 500 mg/kg three times weekly, at 2–3-day intervals, for six weeks (18 administrations). The control group received an equal volume of normal saline intraperitoneally (Shijiazhuang No. 4 Pharmaceutical Co., Ltd., Shijiazhuang, China). At eight weeks after ligation, animals were euthanized and maxillae were collected.

### 4.6. Micro-CT Scanning and Analysis

Micro-CT scanning was performed using a SkyScan 1276 system (Bruker, Billerica, MA, USA). A total of 12 implants were included in the analysis, comprising six implants from the control group and six from the 2-DG group. One additional implant from the 2-DG group was excluded because it was inadvertently unscrewed during specimen harvesting. Images were acquired at 70 kV and 114 µA with an isotropic voxel size of 18 µm. Standardized mesiodistal, buccolingual, and cross-sectional reconstructions were generated for subsequent evaluation.

Quantitative analysis was performed using Mimics (Materialise, Leuven, Belgium). Bone resorption was measured at the mesial, distal, buccal, and palatal aspects as the distance from the implant platform to the most coronal bone-to-implant contact. To correct for angular distortion, exposed implant height (mm) was calculated as 3.8 × (e/d), where e is the measured exposed length in the section and d is the implant length captured in the same plane (Figure 2A).

The constant 3.8 mm represents the actual implant length. All measurements were performed independently by two investigators blinded to group allocation. Discrepancies were resolved by consensus.

### 4.7. Histological Processing and Analysis

Specimens were fixed in 4% paraformaldehyde (Solarbio, Beijing, China; Cat. No. P1110) for 24 h at room temperature and then decalcified for one month in 10% EDTA decalcifying solution (pH 7.2; Solarbio, Beijing, China; Cat. No. E1171) at 4 °C. The decalcification solution was replaced weekly. After softening, implants were carefully counter-rotated and removed. Specimens were paraffin-embedded, sectioned sagittally at 4 µm, baked at 60 °C, and stored under desiccated conditions before H&E staining and IHC.

For IHC, sections were dewaxed in xylene, rehydrated through graded ethanol, treated with 3% hydrogen peroxide to quench endogenous peroxidase, and blocked with 10% BSA (Amresco, Solon, OH, USA; Cat. No. 0332). Sections were incubated with rabbit monoclonal anti-FOXP3 antibody (1:100; eBioscience, San Diego, CA, USA; 14-5773-80), rabbit polyclonal anti-IL-17A antibody (1:600; Abcam, Cambridge, UK; ab214588), rabbit monoclonal anti-iNOS antibody (1:4000; Abcam; ab178945), or rabbit polyclonal anti-CD206 antibody (1:1000; Abcam; ab64693). After incubation with horseradish-peroxidase-conjugated goat anti-rabbit IgG (Gene Tech, Shanghai, China; GK500610A) at 37 °C for 30 min, staining was developed with DAB (Gene Tech, Shanghai, China; GK600710) and nuclei were counterstained with hematoxylin (Gene Tech, Shanghai, China; GT100540).

Images were acquired using an Aperio AT2 digital slide scanner (Leica Biosystems, Wetzlar, Germany) and evaluated with SlideViewer (version 2.5; 3DHISTECH Ltd., Budapest, Hungary). For epithelial integrity, specimens with intact epithelium on both mesial and distal sides were classified as intact. For epithelial-thickness analysis, specimens with intact epithelium on at least one side were included (*n* = 3 per group); the epithelial area within 1 mm of the implant margin was measured in Fiji ImageJ (version 1.54p), and average thickness was calculated from area/reference length. ICT area was measured by outlining the inflammatory infiltration zone in mesial and distal peri-implant soft tissue. IHC images were selected from corresponding regions in consecutive sections and assessed qualitatively; no cell-count or staining-intensity quantification was performed.

### 4.8. Reverse Transcription-Quantitative PCR

Peri-implant soft tissue was collected from three biological samples per group, immersed in RNAlater (Invitrogen, Carlsbad, CA, USA; Cat. No. AM7020), and stored at −80 °C. Total RNA was isolated with TRIzol (Sigma-Aldrich, St. Louis, MO, USA; Cat. No. T9424). cDNA was synthesized using the Hieff III 1st Strand cDNA Synthesis Kit (Yeasen, Shanghai, China; Cat. No. 11139ES) according to the manufacturer’s instructions. RT-qPCR was performed using Hieff qPCR SYBR Green Master Mix (Yeasen, Shanghai, China; Cat. No. 11201ES). Gene expression was normalized to GAPDH and calculated using the 2^−ΔΔCt^ method. Primer sequences are listed in Table S1. Because RNA was extracted from whole peri-implant soft tissue, the results represent tissue-level expression and cannot be assigned to a specific immune-cell population.

### 4.9. Statistical Analysis

Statistical analyses were performed using GraphPad Prism 8 (GraphPad Software, San Diego, CA, USA), and Fiji ImageJ was used for image measurements. Normally distributed continuous variables were compared between two independent groups using unpaired two-tailed Student’s *t*-tests (Figure 2C, Figure 3F,I, Figure 4A and Figure 5A). Ordinal/categorical epithelial-integrity data were compared using the Mann–Whitney U test (Figure 3C). Associations involving non-normally distributed or ordinal variables were assessed using Spearman correlation (Figure 3J,K). Data are presented as mean ± SD where applicable. Statistical significance was defined as *p* < 0.05; * *p* < 0.05, ** *p* < 0.01, *** *p* < 0.001; ns, not significant.

## 5. Conclusions

In summary, systemic 2-DG treatment attenuated peri-implant inflammatory changes and bone resorption in experimental peri-implantitis, accompanied by reduced expression of multiple M1- and Th17-associated inflammatory markers. These findings suggest that 2-DG may modulate innate- and adaptive-immunity-associated inflammatory responses within the peri-implant microenvironment and support further investigation of glycolysis-targeted immunometabolic modulation as an adjunctive therapeutic strategy for peri-implantitis.

## Data Availability

The original contributions presented in this study are included in the article/Supplementary Material. Further inquiries can be directed to the corresponding authors.

## Supplementary Materials

The following supporting information can be downloaded at https://www.mdpi.com/article/10.3390/ijms27177513/s1.

## Abbreviations

The following abbreviations are used in this manuscript:

2-DG	2-deoxy-D-glucose
BIC	bone-to-implant contact
H&E	hematoxylin and eosin
ICT	infiltrated connective tissue
IHC	immunohistochemistry
IM	implant space
RT-qPCR	reverse transcription-quantitative PCR
