# Supplementary material for: Targeting Glycolysis May Bridge Innate and Adaptive Immune Regulation in Experimental Peri-Implantitis
Source: Int J Mol Sci. 2026 Aug 22;27(17):7513. doi: 10.3390/ijms27177513 (PMC13566337; doi:10.3390/ijms27177513)
Supplement: Supplementary file 1 [file ijms-27-07513-s001.zip › Table S1.pdf]

Table S1. Primer sequences of related genes for rat M1/M2 macrophages and Th17/Treg lymphocytes.

Gene	Sequence
rat-GAPDH	Forward 5'-ACTCCACGACGTACTCAGCG-3'
	Reverse 5'-GGTCGGAGTCAACGGATTTG-3'
rat-CD86	Forward 5'-TAAGCAAGGTCACCCGAAAC-3
	Reverse 5'-AGCAGCATCACAAGGAGGAG-3'
rat-iNOS	Forward 5'-GGATCTTCCCAGGCAACCA-3'
	Reverse 5'-AATCCACAACTCGCTCCAAGATT-3'
rat-CCR7	Forward 5'-CAGGTGTGCTTCTGCCAAGAT3'
	Reverse 5'-GGTAGGTATCCGTCATGGTCT-3'
rat-IL-1β	Forward 5'-TCTGTGACTCGTGGGATGAT-3’
	Reverse 5'-CTTCTTTGGGTATTGTTTGG-3'
rat-TNF- α	Forward 5'-GGATCTCAAAGACAACCAAC-3°
	Reverse 5'-ACAGAGCAATGACTCCAAAG-3'
rat-IL-6	Forward 5'-ACAGTGCATCATCGCTGTTC-3'
	Reverse 5'-CCGGAGAGGAGACTTCACAG-3'
rat-IL-18	Forward 5'-CAACCGCAGTAATACGGAGC-3'
	Reverse 5'-TCTGGTCTGGGATTCGTTGG-3'
rat-CD163	Forward 5'-GTTCCCTGCATAGCAAGTGG-3'
	Reverse 5'-CCGAAGTAAGCAGAGCCAGT-3'
rat-Arg-1	Forward 5'-GCATCCACGTCGTTCAAAACA-3'
	Reverse 5'-GTTATCTCTGTGCCCTGTGGG-3'
rat-IL-17	Forward 5'-CCATCCATGTGCCTGATGCT-3'
	Reverse 5'-AAGTTATTGGCCTCGGCGTT-3'
rat-RORγt	Forward 5'-AGTGTAATGTGGCCTACTCCT-3
	Reverse 5'-GCTGCTGTTGCAGTTGTTTCT-3'
rat-IL-21	Forward 5’-TGATGTCTGCCTGTGACCTGTG-3’
	Reverse 5’-CCAGAGGTAGTCAGTGTAGCAGGTG-3’
rat-TGF-β	Forward 5’-CTGCTGACCCCCACTGATAC-3’
	Reverse 5’-AGCCCTGTATTCCGTCTCCT-3’
rat-FOXP3	Forward 5’-CCCAGGAAAGACAGCAACC-3’
	Reverse 5’-TTCTCACAACCAGGCCACTTG-3’
rat-IL-10	Forward 5’-GCCAAGCCTTGTCAGAAATGA-3’
	Reverse 5’-TTTCTGGGGCCATGGTTCTCT-3’
